# Clinical overlap between at-risk mental states and attention-deficit/hyperactivity symptoms in community adolescents: investigating the influence of sex

**DOI:** 10.3389/fpsyt.2026.1872849

**Published:** 2026-09-23

**Authors:** Francisca Castellano-García, Antonio Jovani, Balma Moliner-Castellano, Rita Gimeno, Ana Benito, Gonzalo Haro

**Affiliations:** 1 TXP Research Group, Cardenal Herrera-CEU University, CEU Universities, Castellón, Spain; 2 Department of Education Sciences, Universidad Cardenal Herrera-CEU, CEU Universities, Castellón, Spain; 3 Private Centre Specialised in Neurodevelopmental Disorders, Castellón, Spain; 4 Department of Mental Health, La Fe University and Polytechnic Hospital, Valencia, Spain; 5 Department of Mental Health, Provincial Hospital Consortium of Castellon, Castellón, Spain; 6 Torrente Mental Health Unit, General University Hospital of Valencia, Torrente, Spain

**Keywords:** adolescents, attention-deficit/hyperactivity disorder (ADHD), clinical high risk, early intervention, early psychosis, prevention, sex differences

## Abstract

**Background:**

Adolescence is a period of heightened vulnerability to mental disorders, including attention-deficit hyperactivity disorder (ADHD) and at-risk mental states for psychosis (ARMS). Although evidence suggests an association between these conditions, research in community adolescent samples is limited, and the moderating role of sex remains unclear. It is also unknown whether the sex-related symptom patterns typically observed in ADHD are preserved in adolescents with ADHD–ARMS.

**Methods:**

A cross-sectional study included 697 non-help-seeking adolescents aged 11–15 years from a community sample of school-attending youth. Validated instruments assessed ADHD symptoms, anxiety, depression, and attenuated psychotic symptoms. Participants were classified into control, low-risk, intermediate-risk, and ARMS groups. Generalized ordinal logistic regression and consistent partial least squares structural equation modeling (PLS-SEM) were used to examine the association between ADHD symptoms and ARMS and the potential moderating effect of sex.

**Results:**

2% of participants exceeded the cut-off for inattentive ADHD symptoms and 18.4% for the combined presentation, while 2.8% were classified as ARMS. A combined presentation of ADHD symptoms was substantially more prevalent in the ARMS group (68.4%) vs controls (6.6%). Hyperactivity–impulsivity predicted ARMS status, particularly in females. In girls, ARMS was associated with hyperactivity–impulsivity and emotional problems, whereas in boys it was associated with depressive symptoms and suicidal ideation. PLS-SEM indicated that sex did not moderate the association between ADHD symptoms and ARMS but had a direct effect on ARMS.

**Conclusions:**

ADHD symptoms meeting the threshold for the combined presentation are associated with ARMS in adolescents. Female sex was associated with higher ARMS scores linked to hyperactivity–impulsivity, while in males risk was related to depression and suicidal ideation. ARMS–symptom associations were stronger in females. These findings support sex-sensitive approaches for early prevention of psychosis risk in youth.

## Introduction

The incidence of mental disorders in adolescents has increased in the last decade because of neurobiological, hormonal, and social factors (1). During adolescence, brain plasticity, changes in the prefrontal cortex, development of the dopaminergic system (2), and the hormonal changes of puberty (3) increase susceptibility to anxiety, depression, and other psychopathologies, especially in women (1).

Attention-deficit hyperactivity disorder (ADHD) is one of the most studied disorders in adolescents, both for its impact on neurodevelopment and its relationship with other mental health problems. Its core symptoms are characterized by persistent inattention and/or hyperactivity and impulsivity, which negatively affect development or functional performance (4). The prevalence of ADHD is 7.47% (5, 6) in the child–adolescent population and 3% in the adult population (7). The presentation of ADHD varies between sexes, predominating in men during adolescence (10.6% vs. 5.28%), although the ratio balances out in adulthood (8). Although there is literature stating that some subtypes of ADHD may be more prevalent in women (9), meta-analytic information indicates that all subtypes (inattentive, hyperactive-impulsive, and combined) are more frequent in men (5).

ADHD has a high prevalence of psychiatric comorbidities such as psychotic disorders that complicate its management (10, 11). Meta-analytic evidence shows that individuals with ADHD in childhood have a 5-fold greater risk of developing a psychotic disorder compared to those who do not have ADHD (12), leading to the hypothesis that ADHD may be an early indicator of psychotic risk. In fact, 23% of patients with schizophrenia reported ADHD symptoms in childhood and adulthood, compared to 2.5% in the general population (13). The theories proposed to explain this relationship include a polygenic contribution and shared perinatal complications between patients with childhood ADHD and adult schizophrenia (12, 14). According to DSM-IV criteria, the prevalence of ADHD subtypes in individuals diagnosed with psychotic disorders is 15% (inattentive), 10% (hyperactive-impulsive), and 15% (combined) (15).

Furthermore, evidence from pediatric and adolescent populations suggests that ADHD is associated with psychotic experiences and psychosis-spectrum symptoms before the onset of psychotic disorders, supporting a potential link between ADHD-related traits and early psychosis vulnerability during neurodevelopment (16, 17). Importantly, developmental studies indicate that the prevalence and clinical significance of psychosis-risk symptoms differ across childhood and adolescence, highlighting the need for age-specific investigations in ARMS populations (18).

At-risk mental state (ARMS) constitutes a prodromal state prior to the onset of frank psychotic symptoms (19). Although the predictive validity of the ARMS construct is higher in help-seeking populations, its assessment in community-based adolescents remains valuable for understanding early psychosis-risk trajectories (20). ADHD is a frequent comorbidity among individuals with ARMS, with prevalence estimates of around 20% in the general ARMS population (21). These ratios increase in the adolescent population, reaching 45% (22). ARMS youth with ADHD have shared demographic and clinical characteristics (21): family history of psychotic disorders, less formal education, greater difficulties in functioning and social cognition, and an early transition to psychosis. However, there is no consensus regarding gender differences in the ADHD–ARMS population, with some studies finding no differences (23) and others indicating its more frequent diagnosis among male patients (21). Similarly, although the clinical presentation of anxiety, symptoms of depression, and suicidal ideation is higher in ARMS individuals (24), studies report disparate prevalences in the ADHD+ARMS population. Finally, although it has been established that the combined subtype in childhood is related to psychotic symptoms in adolescence (14), no studies have been identified that specifically address ADHD subtypes in the ARMS population.

Evidence exploring the underlying mechanisms in the relationship between ARMS and ADHD is scarce. Recent evidence from ARMS populations indicates that approximately 20% of young people meeting ARMS criteria also fulfil criteria for ADHD and present poorer role functioning, premorbid adjustment, and social cognition compared with those without ADHD, although no differences have been observed in attenuated psychotic symptoms or transition to psychosis (21). Although mediators between ADHD and psychosis such as substance use, impulsivity (25), and bullying (16), have been addressed, it remains unclear whether sex plays a role or acts as a mediator or moderator in this relationship. The available evidence indicates that women with ADHD tend to present internalizing symptoms such as anxiety, depression, and suicidal ideation (26) while externalizing symptoms predominate in men, especially impulsive behaviors and motor hyperactivity (21). However, it is unknown whether this differential sex distribution persists in adolescents with ADHD who present ARMS, highlighting the importance of investigating how symptom profiles relate to the risk of psychosis in this population. Previous studies have focused on the adult clinical population with schizophrenia spectrum disorders, while to the best of our knowledge, no article to date has focused on the general adolescent population with ARMS and ADHD. Since psychotic symptoms are a common comorbidity in people with ADHD, understanding this association is critical to improving diagnosis and clinical intervention, especially during adolescence—a key stage for detecting risk factors.

Given all the above, the objectives of this article were (a) to evaluate whether the presence of ADHD symptoms increases the risk of ARMS; (b) to determine the prevalence of ADHD subtypes in ARMS individuals, and (c) to examine if sex has a moderator and/or direct effect in the presence of ARMS in patients with ADHD symptoms.

## Materials and methods

### Study type and sampling technique

This was an observational, cross-sectional analytical study. The sample size was calculated with Epidat 3.1 (27) to evaluate the prevalence of ARMS (1.7% in the general adolescent population) (5) with an accuracy of 1%. Considering the population of Castellón aged 11 to 15 years (*N* = 8,249) as the reference (28), we concluded that a sample of 596 would be necessary. Likewise, G*Power software (version 3.1.9.4) was used to calculate that the sample size required for multivariate analysis of variance (MANOVA) would be 296 participants when applying an effect size 0.02, power of 0.80, and alpha of 0.05, with 4 groups and 4 variables. According to Green’s tables (29), to model the data with four predictors, small effect size, power of 0.80 and alpha of 0.05, the required sample size would be 599. Non-random sampling was carried out on adolescents from the general population aged between 11 and 15 years without a clinical history of ARMS or ADHD at 9 high schools in the city of Castellón, Spain. A total sample of 697 students in their first and second years of compulsory secondary education (ESO) was recruited.

### Inclusion and exclusion criteria

The inclusion criteria established that participants had to be part of the first cycle of ESO (1st and 2nd years), corresponding to ages between 11 and 15 years, and had to have an adequate level of Spanish language skills. Students with special educational needs were excluded.

### Assessment instruments

The psychometric tests employed were:

- The Prodromal Questionnaire-Brief (PQ-B), comprising 21 items formulated in a dichotomous (true/false) format to assess prodromal symptoms of positive-type psychosis (30). In terms of the psychometric properties of the PQ-B, the test presented an ordinal alpha of 0.95 (31).
- The Youth Psychosis At-Risk Questionnaire Brief (YPARQ-B) assesses self-reported subclinical psychotic experiences in adolescents in the general population and was developed specifically to screen for ARMS symptoms during adolescence (32). The internal consistency of the total YPARQ-B score was 0.94.
- The Attention Deficit-Hyperactivity Disorder Rating Scale IV (ADHD-RS-IV) (33) consists of an inattention subscale (9 items) and a hyperactivity/impulsivity subscale (9 items), with a total of 18 items. It has a sensitivity of 91.9% and a specificity of 90.3%.
- The Patient Health Questionnaire 9 for Adolescents (PHQ9-A) (34) is the most reliable self-administered questionnaire for assessing the clinical presence of depression in adolescents, with a sensitivity and specificity of 86.2 and 82.9% respectively (35).
- The State-Trait Anxiety Inventory for Children (STAI-C) is the adaptation of the STAI questionnaire for children aged 8 to 15 years (36). It contains 20 elements intended to evaluate the anxiety level of the child at a given time (state anxiety), while the second aims to identify how the subject feels in general (trait anxiety) through another 20 elements.
- The Adolescent Suicidal Behavior Assessment Scale (SENTIA) is composed of 16 items in a dichotomous format that assesses all spectrums of suicidal behavior: ideation, planning, intention, communication, and action (37). It has adequate psychometric properties: Omega coefficient = 0.95.
- The Strengths and Difficulties Questionnaire (SDQ) (38) assesses five domains: emotional symptoms, behavioral problems, hyperactivity/inattention, peer problems, and prosocial behavior. The ordinal alpha and omega coefficients provided good estimates in all subscales of this questionnaire (ranging from 0.70 to 0.92).
- Comprehensive Assessment of At Risk Mental States (CAARMS) individual interview. This semi-structured clinical interview assesses incipient positive symptoms, sensory-perceptive alterations, and thought and language disorders. Each item is rated on a scale of 1–6, where 3 is the cut-off point for ARMS and 6 corresponds to first psychotic episode. The CAARMS is a precise instrument with an area under the curve (AUC) of 0.85 (95% CI [0.81, 0.88]) and a high sensitivity of 0.93 (95% CI [0. 87, 0.96]), although it has a lower specificity of 0.58 (95% CI [0.50, 0.66]) (20). This interview was conducted by two researchers who also work as clinicians and have training and experience in its use.
- Neurodevelopment in Adolescence-Parents 1 (NA-P1) questionnaire. Our group created this test, which encompasses sociodemographic data (grade, age, and academic performance, among others) as well as information related to mental health history. This questionnaire was completed by the participants’ parents.

### Procedure

First, the directors of the educational centers were contacted. The sample was then recruited between September and November 2022. During these months, self-administered questionnaires were individually completed by the adolescents during school hours, which required approximately 60–90 minutes. This phase was supported by four researchers, who were trained in the tests used. In addition, the parents of the participants completed the NA-P1.

The PQ-B and YPARQ-B were used as first-stage screening instruments for psychotic-like experiences and psychosis risk. Participants scoring above the cut-off on both measures underwent a CAARMS interview in May and June of 2023. The combined use of both questionnaires aimed to increase screening specificity, reduce false positives, and follow a stepped assessment approach recommended for community samples. The agreement between both instruments was evaluated in the sample: r = 0.774 (p < 0.001), ICC = 0.740 (p < 0.001), % agreement = 65.8%, and kappa = 0.168 (p < 0.001). It is worth noting that the difference between the percentage of agreement and kappa is due to the fact that YPARQ-B classifies fewer cases as positive (46/688, 6.7%) than PQ-B (273/688, 39.7%).

As was done in a previous publication by our group (39), participants were classified into four groups: (1) healthy controls, scoring below the cut-offs on both the PQ-B and YPARQ-B (n = 413; 59.3%); (2) low-risk, exceeding the cut-off on only one screening measure (n = 235; 34.1%); (3) intermediate-risk, exceeding both cut-offs but not meeting CAARMS criteria for ARMS (n = 23; 3.2%); and (4) at-risk (ARMS), exceeding both cut-offs and meeting CAARMS criteria, representing the highest-risk group along the psychosis-risk continuum (n = 19; 2.8%). The term ARMS was reserved for the risk group confirmed by CAARMS, whereas the low- and intermediate-risk groups were based on screening measures. These groups were defined for analytical purposes and do not represent diagnostic categories. **Figure 1** shows the sample selection process and its division into the four study groups. This approach was based on previous studies examining psychosis risk along a continuum (40).

**Figure 1:**
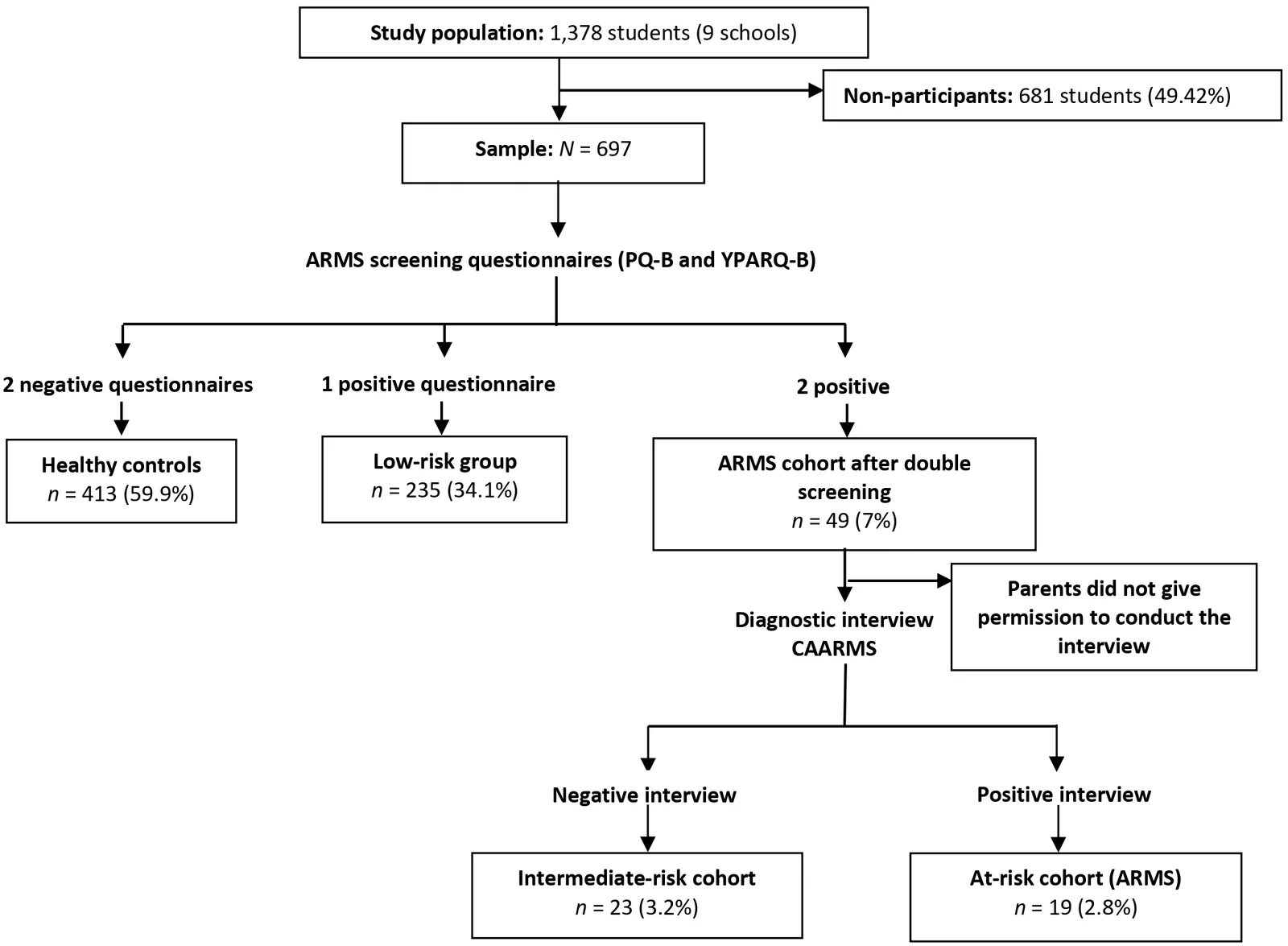
Flowchart showing the process for diagnosing the at-risk mental state (ARMS). CAARMS, The Comprehensive Assessment of At-Risk Mental States.

### Statistical analysis

Data were analyzed using SPSS software for Microsoft (version 29, IBM Corp., Armonk, NY). After the exploratory and descriptive analysis and verification that the statistical assumptions were met, the relationships between the variables evaluated were studied using Chi squared tests for categorical variables, correlations for quantitative variables, and MANOVA and subsequent analysis of variance (ANOVA) tests to analyze the differences between the study groups (healthy controls, low-risk, intermediate-risk, and ARMS) for the quantitative variables. Given the difference in sample size between the different study cohorts, Bonferroni correction was used. For each variable that showed differences between the studied groups, ordinal logistic regression was performed using unadjusted generalized linear models, for exploratory purposes, to use those that showed predictive power in the ordinal logistic regression model adjusted by the rest of the variables to try to predict inclusion in each of the groups studied. The group variable to which the participant belonged was considered ordinal, given that each group had a higher ARMS score than the previous one. ADHD variables containing redundant information were not included as predictors in order to avoid collinearity problems. Likewise, the data was modelled using SmartPLS (41) to assess relationships between ARMS, ADHD, and sex. Consistent partial least squares structural equation modeling (PLS-SEM) with bootstrapping and one-tailed Student’s t-test was used. All analyses were performed on the total sample, including the four groups studied (controls, low-risk, intermediate-risk, at-risk of psychosis).

### Ethical considerations

The project was authorized by the Ethics Committee for Drug Research (CEIM) at the Castellón Provincial Hospital Consortium (CEIM-41-3), by the Ethics Committee for Biomedical Research of the Cardenal Herrera-CEU University (CEI22/335), and the Department of Education, Culture and Sport of the Valencian Community (CSV: EMER9QZU:M27XBBIB:91AKUXLY). Given that all participants were minors aged 11–15 years, written informed consent was obtained from their legal guardians (their parents in all cases), and the adolescents provided their assent to participate. The informed consent procedure was conducted in accordance with Spanish legislation on patient autonomy and informed consent (Law 41/2002, of 14 November).

## Results

### Sociodemographic and clinical characteristics related to psychosis risk groups

**Table 1** summarizes the sociodemographic and clinical characteristics of the sample across the four study groups: healthy controls, low-risk, intermediate-risk, and at-risk (ARMS). The participants were predominantly female (51.7%), with a mean age of 12.6 years (SD = 0.7). Approximately half were enrolled in the first (50.7%) and second (49.3%) years of secondary education. Most attended public (57.4%) or charter schools (40.1%). The majority showed average to good academic performance (70.2%), and 19.2% had previously received mental health care. No significant group differences were observed for most sociodemographic variables, except for sex, with more boys in the control group and more girls in the low-risk group.

**Table 1:** Sociodemographic and clinical characteristics of the adolescents included in this study.

Overall sample %/Mean (n/SD)	Controls %/Mean (n/SD)	Low-risk %/Mean (n/SD)	Intermediate-risk %/Mean (n/SD)	At-risk (ARMS) %/Mean (n/SD)	χ^2/F p-value	Effect size (ES) Corrected typified residuals (CTR)
Sex	Male	48.3% (333)	**53.5% (221)**	**40.6% (95)**	39.1% (9)	42.1% (8)	11.11 0.011*	**0.127 3.3/−2.9 −3.3/2.9**
	Female	51.7% (356)	**46.5% (192)**	**59.4% (139)**	60.9% (14)	57.9% (11)		
Age (years)		12.6 (0.7)	12.6 (0.7)	12.6 (0.7)	12.6 (0.4)	12.6 (0.7)	0.25 0.860	0.001
High-schools	Public	57.4% (396)	57.4% (237)	57.9% (136)	47.8% (11)	63.2% (12)	3.13 0.792	0.048
	Charter (state-subsidized)	40.1% (277)	40.7% (168)	38.7% (91)	47.8% (11)	36.8% (7)		
	Private	2.5% (17)	1.9% (8)	3.4% (8)	4.3% (1)	0% 0		
School year	1st year ESO	50.7% 350	49.6% (205)	51.5% (121)	60.9% (14)	52.6% (10)	1.22 0.747	0.042
	2nd year ESO	49.3% (340)	50.4% (208)	48.5% (114)	39.1% (9)	47.4% (9)		
Academic performance	Fail	5.9% (33)	4.3% (14)	8.7% (17)	5% (1)	6.7% (1)	11.09 0.269	0.082
	Pass	25.9% (144)	23.8% (77)	28.1% (55)	25% (5)	46.7% (7)		
	Exceptional	44.3% (246)	45.7% (148)	43.4% (85)	40% (8)	33.3% (5)		
	Outstanding	23.8% (132)	26.2% (85)	19.9% (39)	30% (6)	13.3% (2)		
Prior mental health monitoring	No	80.8% (451)	82% (269)	78.5% (153)	85% (17)	80% (12)	1.23 0.753	0.047
	Yes	19.2% (107)	18% (59)	21.5% (42)	15% (3)	20% (3)		
Psychiatric medication (amphetamine derivatives)	No	97.1% (543)	96.3% (315)	98.5% (194)	100% (20)	93.3% (14)	3.40 0.333	0.078
	Yes	2.9% (16)	3.7% (12)	1.5% (3)	0% 0	6.7% (1)		
Mental disorder	No	89% (614)	91% (376)	86% (202)	87% (20)	84.2% (16)	4.51 0.204	0.081
	Yes	11% (76)	9% (37)	14% (33)	13 (3)	15.8% (3)		
Substance consumption	No	97.5% (673)	**99% (409)**	**95.3% (224)**	100% (23)	**89.5% (17)**	14.37 0.015*	**0.144 3.1/−2.7/−2.3 −3.1/2.7/2.3**
	Yes	2.5% (17)	**1% (4)**	**4.7% (11)**	0% 0	**10.5% (2)**		
Subjective parental assessment of ADHD	Never	60.6% (335)	63.1% (200)	59% (115)	42.1% (8)	50% (7)	6.4 0.383	0.076
	Maybe	22.1% (122)	21.8% (71)	22.1% (43)	26.3% (5)	21.4% (3)		
	Yes	17.4% (96)	15.1% (49)	19% (37)	31.6% (6)	28.6% (4)		
Needs mental health care	No	83% (459)	**87.1% (283)**	79% (154)	68.4% (13)	64.3% (9)	12.90 0.048*	**0.108 3 −2.7**
	Maybe	10.3% (57)	**7.4% (24)**	13.3% (26)	21.1% (4)	21.4% (3)		
	Yes	6.7% (37)	5.5% (18)	7.7% (15)	10.5% (2)	14.3% (2)		
Family history of ADHD	No	95.3% (529)	95.7% (311)	95.9% (188)	85% (17)	92.9% (13)	5.21 0.141	0.97
	Yes	4.7% (26)	4.3% (14)	4.1% (8)	15% (3)	7.1% (1)		

Significant differences are highlighted in bold. *p < 0.05; **p < 0.01; ***p < 0.001.

Regarding ADHD-related variables, 4.7% reported a family history of ADHD and 2.9% were receiving stimulant treatment, with no significant differences between groups. Parents reported attention or impulsivity symptoms in 40% of adolescents, without group differences.

Significant group differences emerged in substance use, bullying, and perceived need for mental health care, with higher prevalence in the intermediate-risk and ARMS groups compared with controls.

### Sex differences in ADHD symptoms and emotional problems

**Table 2** shows the psychopathological differences between male and female participants. Significantly higher scores were observed in the total ADHD symptoms score (including inattentive and combined symptom presentations) in males. However, no differences were found between males and females for the cut-off points for inattentive/combined ADHD symptoms.

**Table 2:** Sex differences in ADHD symptoms variables and anxiety–depressive disorder symptoms.

		Overall sample Average/% (*SD*/*n*)	Men Average/% (*SD*/*n*)	Women Average/% (*SD*/*n*)	*F*/*χ*^2^*p*-value	Effect size (ES) significant differences/CTR
Total inattention symptoms score (ADHD RS)			**7.30 (4.88)**	**7.28 (5.82)**	10.8 0.0016**	**0.410**
Total hyperactivity-impulsivity symptoms score (ADHD RS)			7.5 (5.03)	7.25 (5.19)	0.350 0.554	0.393
Total combined ADHD symptoms score (ADHD RS)			**14.8 (9.21)**	**14.53 (10.42)**	6.402 0.012*	**0.753**
Inattentive ADHD symptoms cut-off point (ADHD RS)	No	98% (675)	99.1% (329)	96.9% (349)	4.14 0.079	0.078
	Yes	2% (14)	0.9% (3)	3.1% (11)		
Combined ADHD symptoms cut-off point (ADHD RS)	No	81.6% (562)	81.9% (272)	81.2% (289)	0.290 0.866	0.021
	Yes	18.4% (127)	18.1% (60)	18.8% (67)		
Emotional problems (SDQ)			**2.39 (2.14)**	**4 (2.71)**	26.250 < 0.001***	**0.187**
Conduct problems (SDQ)			3.24 (1.52)	3.12 (1.46)	1.82 0.177	0.115
Prosocial behavior (SDQ)			**8.08 (1.84)**	**8.48 (1.63)**	4.57 0.033*	**0.132**
Suicidal ideation (SENTIA, summary)			**0.85 (2.38)**	**2.23 (3.34)**	69.922 < 0.001***	**0.218**
Depression (PHQ-A)			**4.67 (4.43)**	**7.21 (6.21)**	54.316 < 0.001***	**0.416**
Anxiety (STAI-C Trait)			**32.12 (6.94)**	**38.38 (9.29)**	47.088 < 0.001***	**0.623**
RISK GROUPS	Controls	59.9% (413)	**66.4% (221)**	**53.9% (192)**	13.046 0.042*	0.097 3.4/−3.3 −3.0/2.9
	Low-risk	34.1% (235)	**28.5% (95)**	**39% (139)**		
	Intermediate-risk	3.3% (23)	2.7% (9)	3.9% (14)		
	At-risk (ARMS)	2.8% (19)	2.4% (8)	3.1% (11)		

Significant differences are highlighted in bold. *p < 0.05; **p < 0.01; ***p < 0.001. CTR, corrected typified residuals; ADHD RS, ADHD Rating Scale IV; SDQ, Strengths and Difficulties Questionnaire; SENTIA, Adolescent Suicidal Behavior Assessment Scale; PHQ-A, Patient Health Questionnaire 9 for Adolescents; STAI-C, State-Trait Anxiety Inventory Children.

In females, there was a greater tendency to present symptoms of depression (PHQ-A = 7.21 vs. 4.67), anxiety (STAI-C = 38.38 vs. 32.12), and emotional problems (SDQ of 4 vs. 2.39), despite having greater prosocial behavior scores (SDQ = 8.48 vs. 8.08). Finally, sex differences were also observed between the control and low-risk groups.

### ARMS and ADHD symptoms

**Table 3** shows a clear gradient of increasing ADHD symptoms across psychosis risk groups. Total ADHD scores (for the combined symptom presentation) rose progressively from controls (M = 10.85) to low (M = 19.23), intermediate (M = 25.70), and ARMS (M = 27.42). Only 2% of participants exceeded the cut-off for inattentive ADHD symptoms, whereas 18.4% exceeded the cut-off for combined ADHD symptoms. Notably, 68.4% of adolescents with ARMS exceeded the cut-off for the combined ADHD symptom presentation compared with 6.6% of controls, indicating a marked concentration of clinically significant ADHD symptoms in the ARMS group.

**Table 3:** Total score and cut-off points for combined inattentive ADHD symptoms (ADHD Rating Scale).

		Overall sample Average/% (*SD*/*n*)	Controls Average/% (*SD*/*n*)	Low-risk Average/% (*SD*/*n*)	Intermediate-risk Average/% (*SD*/*n*)	At-risk (ARMS) Average/% (*SD*/*n*)	*F*/*χ*^2^ *p*-value	Effect size (ES) significant differences/CTR
Total inattention symptoms score		7.29 (5.38)	5.36 4.14	9.52 (5.42)	13.43 (6.82)	13.95 (5.58)	64.82 < 0.001***	**0.235** C<LR, IR and HR LR<IR and HR
Total hyperactivity-impulsivity symptoms score		7.37 (5.11)	5.49 (3.98)	9.71 (5.06)	12.26 (6.42)	13.47 (5.44)	65.261 < 0.001***	**0.230** C<LR, IR and HR LR<HR
Total combined ADHD symptoms score		14.66 (9.84)	10.85 (7.40)	19.23 (9.67)	25.70 (12.59)	27.42 (10.46)	76.721 < 0.001***	**0.263** C<LR, IR and HR LR<IR and HR
Inattentive ADHD symptoms cut-off point	No	98% (675)	**99.8% (411)**	98.3% (231)	**73.9% (17)**	**84.2% (16)**	91.676 < 0.001***	**0.365 4.1/−8.3/4.3 −4.1/8.3/4.3**
	Yes	2% (14)	**0.2% (1)**	1.7% (4)	**26.1% (6)**	**15.8% (3)**		
Combined ADHD symptoms cut-off point	No	81.6% (562)	**93.4% (385)**	**68.1% (160)**	**47.8% (11)**	**31.6% (6)**	116.076 < 0.001***	**0.410 9.8/−6.6/−4.2/−5.7 −9.8/6.6/4.2/5.7**
	Yes	18.4% (127)	**6.6% (27)**	**31.9% (75)**	**52.2% (12)**	**68.4% (13)**		

Significant differences are highlighted in bold. *p < 0.05; **p < 0.01; ***p < 0.001.

### ARMS, emotional problems and prosocial behavior

**Table 4** shows results of clinical variables associated with behavioral problems, anxiety, and depression symptoms in the 4 study groups. The low, intermediate-risk and ARMS groups had higher scores on emotional problems, behavioral problems, and peer problems (SDQ questionnaire). These differences were statistically significant. In addition, less prosocial behavior was found in the ARMS group compared to the other groups. Higher levels of depression, suicidal ideation, and anxiety were also observed in the at-risk groups compared to controls.

**Table 4:** ARMS, emotional problems, and prosocial behavior.

	Overall sample Average/% (*SD*/*n*)	Controls Average/% (*SD*/*n*)	Low-risk Average/% (*SD*/*n*)	Intermediate-risk Average/% (*SD*/*n*)	At-risk (ARMS) Average/% (*SD*/*n*)	*F*/*χ*^2^*p*-value
Emotional problems	3.23 (2.58)	**2.2 (1.98)**	**4.39 (2.44)**	**7.22 (6.16)**	**6.16 (3.11)**	88.012 < 0.001***
Behavioural problems	3.18 (1.49)	**2.87 (1.32)**	**3.49 (1.54)**	**4.57 (1.67)**	**4.26 (1.91)**	20.87 < 0.001***
Problems with peers	4.73 (1.35)	**5.53 (1.28)**	**4.88 (1.34)**	**5.87 (1.18)**	**5.95 (1.84)**	15.26 < 0.001***
Prosocial behavior	8.27 (1.74)	**8.41 (1.74)**	**8.13 (1.68)**	**8.52 (1.150)**	**6.68 (2.11)**	6.88 < 0.001***
Depression (PHQ-A)	5.99 (5.56)	**3.62 (3.85)**	**8.58 (6.99)**	**14.96 (6.99)**	**14.53 (6.56)**	114.51 < 0.001***
Suicidal ideation (SENTIA, summary)	1.56 (2.99)	**0.57 (1.82)**	**2.50 (3.17)**	**5.27 (4.06)**	**7.05 (5.90)**	72.022 < 0.001***
Anxiety (STAI-C trait)	35.37 (8.812)	**31.72 (7.002)**	**39.88 (7.903)**	**46.96 (9.53)**	**45.05 (9.18)**	89.98 < 0.001***

Significant differences are highlighted in bold. *p < 0.05; **p < 0.01; ***p < 0.001.

### Ordinal logistic regression

As can be seen in **Table 5**, most of the variables in which there were differences between the groups allowed the group to be individually predicted. However, when the model adjusted for the remaining variables was evaluated, only the hyperactivity–impulsivity score, emotional problems, depression, and suicidal ideation retained this predictive power. **Tables 6** and **7** show that the predictor variables varied when men and women were considered separately. Specifically, in men, ARMS was predicted by depression and suicidal ideation and in women, it was predicted by hyperactivity–impulsivity and emotional problems.

**Table 5:** Ordinal logistic regression: dependent variable group (control, low-risk, intermediate-risk, at-risk–ARMS) in overall sample.

Variable	Unadjusted model			Adjusted model		
	Exp (B)	95% CI	*p*-value	Exp (B)	95% CI	*p*-value
Sex (female)	**1.658**	**1.224/2.246**	0.001**	0.870	0.557/1.360	0.542
Substance use (Yes)	0.472	0.152/1.446	0.194	0.317	0.065/1.554	0.157
Needs mental health help (Yes)	1.792	0.931/3.452	0.081	0.546	0.243/1.227	0.143
Total inattention symptoms score	**1.211**	**1,172/1,250**	< 0.001***	1.030	0.971/1.092	0.332
Total hyperactivity-impulsivity symptoms score	**1.223**	**1.182/1.264**	< 0.001***	**1.088**	**1.027/1.153**	0.004**
Emotional problems	**1.599**	**1.489/1.717**	< 0.001***	**1.143**	**1.013/1.291**	0.031*
Behavioural problems	**1.466**	**1.319/1.630**	< 0.001***	0.942	0.808/1.098	0.447
Problems with colleagues	**1.398**	**1.241/1.573**	< 0.001***	1.110	0.953/1.294	0.179
Prosocial behavior	**0.883**	**0.810/0.963**	0.005**	0.931	0.824/1.052	0.252
Depression (PHQ-A)	**1.273**	**1.230/1.317**	< 0.001***	**1.069**	**1.002/1.141**	0.044*
Suicidal ideation (SENTIA, summary)	**1.418**	**1.334/1.507**	< 0.001***	**1.124**	**1.028/1.229**	0.010*
Anxiety (STAI-C trait)	**1.154**	**1.130/1.179**	< 0.001***	1.033	0.993/1.075	0.103

Significant results are highlighted in bold. *p < 0.05; **p < 0.01; ***p < 0.001.

**Table 6:** Ordinal logistic regression: dependent variable group (control, low-risk, intermediate-risk, at-risk–ARMS) in men.

Variable	Unadjusted model			Adjusted model		
	Exp (B)	95% CI	*p*-value	Exp (B)	95% CI	*p*-value
Substance use (Yes)	0.507	0.140/1.837	0.301	0.401	0.080/2.008	0.266
Needs help SM (Yes)	1.278	0.451/3.618	0.644	0.759	0.216/2.671	0.143
Total inattention symptoms score	**1.184**	**1.126/1.244**	< 0.001***	1.004	0.912/1.106	0.934
Total hyperactivity-impulsivity symptoms score	**1.192**	**1.135/1.252**	< 0.001***	1.049	0.962/1.144	0.277
Emotional problems	**1.561**	**1.392/1.750**	< 0.001***	1.061	0.890/1.264	0.512
Behavioural problems	**1.397**	**1.204/1.622**	< 0.001***	0.980	0.787/1.220	0.857
Problems with colleagues	**1.229**	**1.034/1.462**	0.019*	1.097	0.876/1.375	0.419
Prosocial behavior	**0.836**	**0.741/0.944**	0.004**	0.867	0.721/1.043	0.130
Depression (PHQ-A)	**1.329**	**1.249/1.414**	< 0.001***	**1.190**	**1.069/1.325**	0.001**
Suicidal ideation (SENTIA, summary)	**1.516**	**1.341/1.713**	< 0.001***	**1.189**	**1.015/1.394**	0.032*
Anxiety (STAI-C trait)	**1.168**	**1.125/1.213**	< 0.001***	1.035	0.975/1.100	0.258

Significant results are highlighted in bold. *p < 0.05; **p < 0.01; ***p < 0.001.

**Table 7:** Ordinal logistic regression: dependent variable group (control, low-risk, intermediate-risk, at-risk–ARMS) in women.

Variable	Unadjusted model			Adjusted model		
	Exp (B)	95% CI	*p*-value	Exp (B)	95% CI	*p*-value
Substance use (Yes)	1.095	0.074/16.212	0.947	< 0.001	0/-	0.999
Needs help SM (Yes)	2.355	0.981/5.656	0.055	0.480	0.158/1.458	0.195
Total inattention symptoms score	**1.240**	**1.187/1.295**	< 0.001***	1.068	0.988/1.155	0.099
Total hyperactivity-impulsivity symptoms score	**1.263**	**1.204/1.324**	< 0.001***	**1.109**	**1.021/1.204**	0.014*
Emotional problems	**1.686**	**1.521/1.868**	< 0.001***	**1.226**	**1.031/1.458**	0.021*
Behavioural problems	**1.615**	**1.384/1.885**	< 0.001***	0.926	0.738/1.161	0.505
Problems with colleagues	**1.570**	**1.332/1.851**	< 0.001***	1.238	0.989/1.551	0.063
Prosocial behavior	0.897	0.790/1.020	0.096	0.984	0.825/1.174	0.856
Depression (PHQ-A)	**1.251**	**1.198/1.306**	< 0.001***	0.997	0.914/1.087	0.944
Suicidal ideation (SENTIA, summary)	**1.381**	**1.282/1.487**	< 0.001***	1.107	0.984/1.244	0.091
Anxiety (STAI-C trait)	**1.165**	**1.130/1.201**	< 0.001***	1.031	0.978/1.088	0.256

Significant results are highlighted in bold. *p < 0.05; **p < 0.01; ***p < 0.001.

### Modeling the relationship between ADHD symptoms and ARMS

To decide which variables to include in the model, a stepwise linear regression was performed on the variables that could predict the ARMS score (Block 1-sex, Block 2-ADHD symptoms, Block 3-emotional problems, depression, and suicidal ideation). The most important predictor was ADHD, with a change in R² of 0.351 and a change in F of 377.43, while depression showed changes of 0.142 and 196.07, emotional problems 0.024 and 34.41, suicidal ideation 0.010 and 14.22, and sex 0.18 and 12.24. Therefore, ADHD symptoms score was included in the structural equation model because it was the main predictor of ARMS, and sex was included because of its relationship with ADHD symptoms and its possible moderating effect on the ADHD-ARMS relationship. That is to say, to evaluate the role of sex in the relationship between ADHD symptoms (items of ADHD-RS-IV) and ARMS (total scores of PQ-B and YPARQ-B), we tested whether sex had a moderating effect in this relationship and/or it could have a direct effect on ARMS.

The model indicated that sex was not a moderator in the relationship between ADHD symptoms and ARMS but rather had a direct effect on ARMS (being female is associated with a higher ARMS score), as shown in **Figure 2**. In this model, all VIFs<3, adjusted R^2^ = 0.46 (p<0.001) and SRMR = 0.059. Since sex influences ARMS, the influence of the variables that were significant in the regression (hyperactivity-impulsivity (ADHD-RS-IV), emotional problems (SDQ), depression (PHQ-A), and suicidal ideation (SENTIA)) on ARMS was modelled separately for both sexes. These models can be seen in **Figures 3** and **4**. In women, all VIFs<3, R^2^ = 0.70 (p<0.001) and SRMR = 0.067. In men, all VIFs<3, R^2^ = 0.64 (p<0.001) and SRMR = 0.076. In these models, the only relationship that maintains significance is that of hyperactivity-impulsivity and ARMS in women, being the path coefficient 0.375 (p<0.001) and the correlation 0.759 (p<0.001), so the explained variance was 28.4%. The effect size of this relationship is f^2^ = 0.186 (moderate).

**Figure 2:**
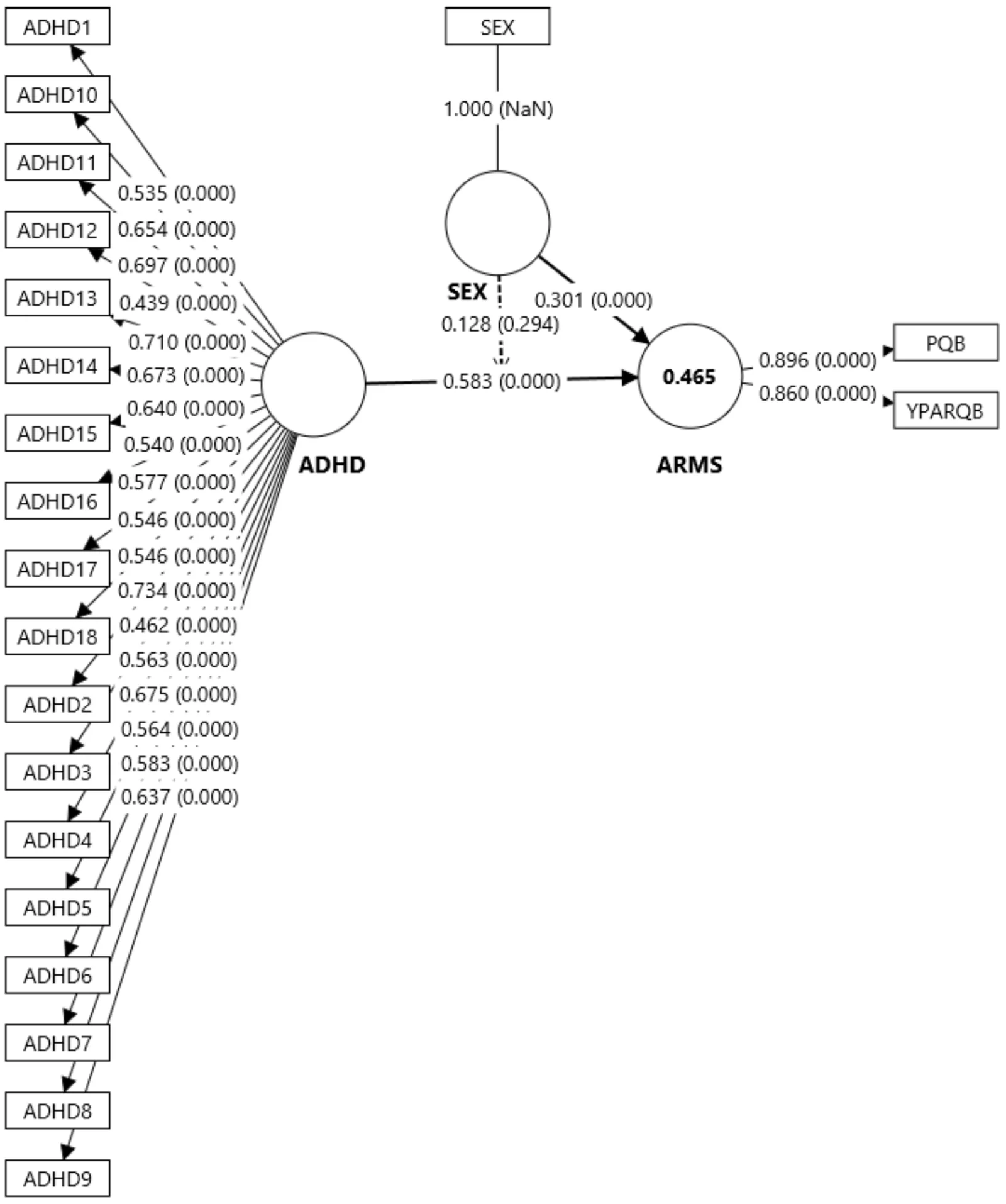
Effect of sex in the relationship between ADHD symptoms (ADHD-RS-IV) and ARMS (PQ-B and YPARQ scores).

**Figure 3:**
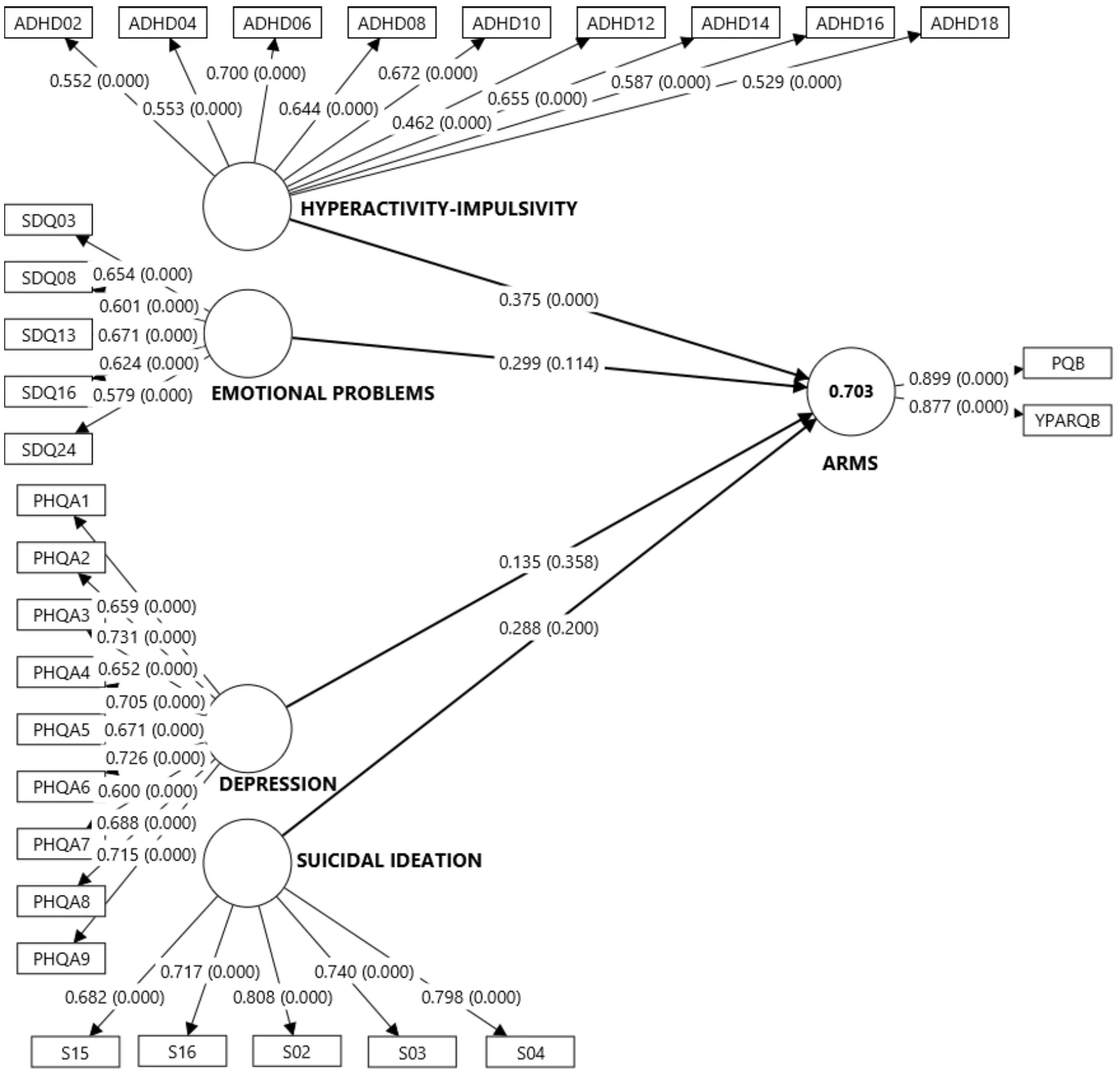
Relationship between hyperactivity-impulsivity symptoms, emotional problems, depression, and suicidal ideation and ARMS (PQ-B and YPARQ scores) in women.

**Figure 4:**
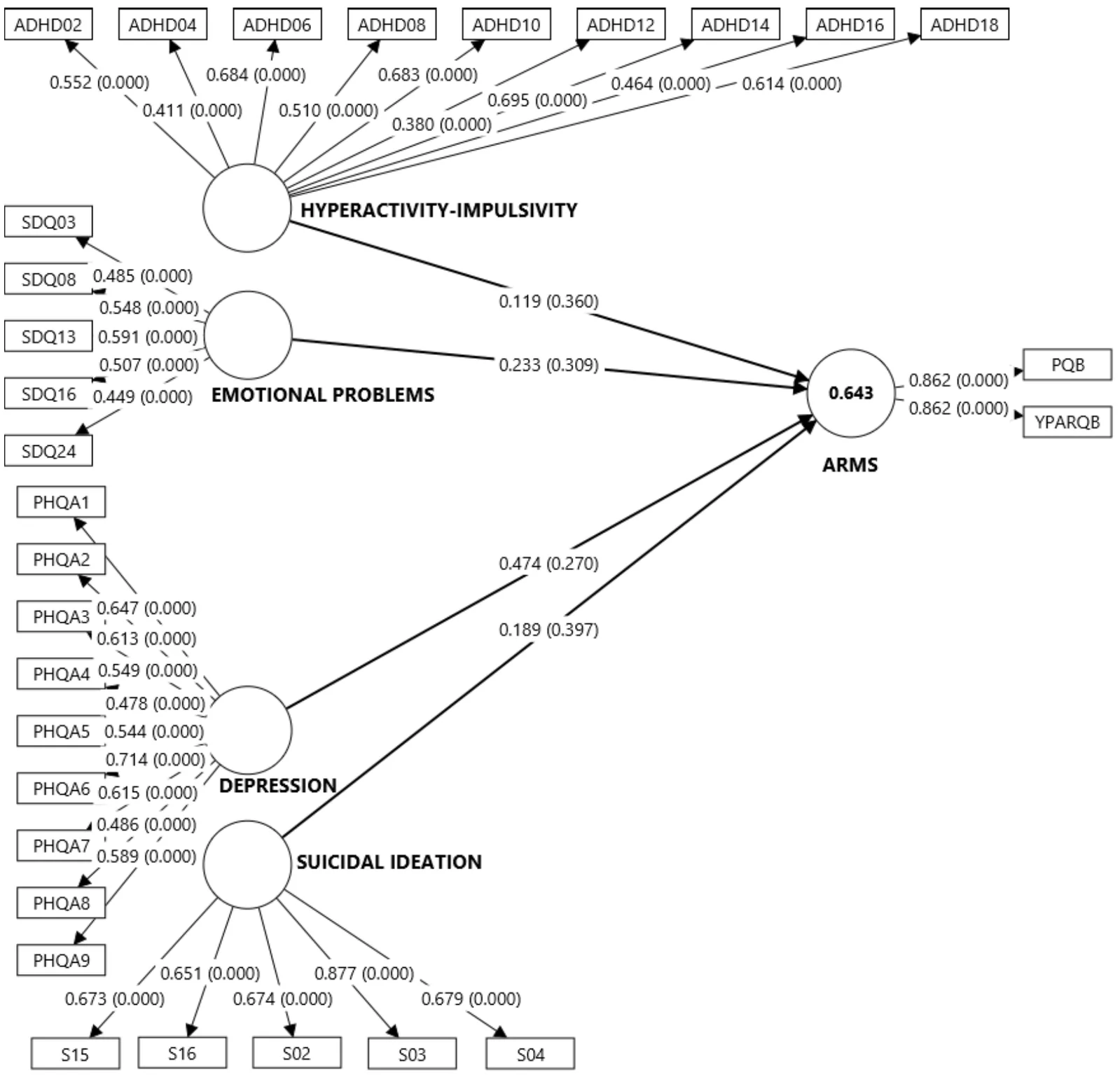
Relationship between hyperactivity-impulsivity symptoms, emotional problems, depression, and suicidal ideation and ARMS (PQ-B and YPARQ scores) in men.

## Discussion

The results of this study provide relevant evidence on the association between ADHD symptoms, particularly symptoms meeting the threshold for the combined presentation, and psychosis-risk states across a continuum of risk (low-, intermediate-, and at-risk/ARMS) in a community-based adolescent sample. Furthermore, distinct sex-specific clinical correlates emerged, with hyperactivity–impulsivity (externalizing symptoms) being more strongly associated with ARMS in girls, whereas depressive symptoms and suicidal ideation (internalizing symptoms) were more relevant in boys. These findings underscore the importance of a multidimensional and sex-sensitive developmental assessment to improve the early identification of adolescents at risk for psychosis and to inform targeted preventive interventions.

First, in our study 2% (*n* = 14) exceeded the cut-off for inattentive ADHD symptoms, compared with 18.4% (*n* = 127) for combined ADHD symptoms. This contrasts with existing evidence that there are no differences between ADHD subtypes in the general adolescent population (5). Furthermore, the numerical difference of around 7% with respect to previous reports is surprising (6), although it depends on the series consulted. Higher overall scores on the ADHD questionnaire were observed in males compared to females and these differences were statistically significant. This difference, supported by previous research (10) is explained both by its attenuated clinical manifestation, as well as by a greater presentation of the inattentive subtype compared to the hyperactive–impulsive subtype (2). However, there were no sex differences in the cut-off scores for inattentive and combined ADHD symptoms. To the best of our knowledge, this is the first study to address the ADHD symptoms and ARMS relationship in a non-clinical population. It was found that ADHD scores increase as the risk of psychosis increases: controls have the fewest ADHD symptoms, and these symptoms progressively increase in the low-, intermediate-, and at-risk (ARMS) groups for psychosis.

In our sample, 34.1% of adolescents (*n* = 235) presented attenuated psychotic symptoms, distributed in different levels of clinical risk: low, intermediate, and at-risk (ARMS). The ARMS subgroup comprised 2.8% of the participants (*n* = 19). The classification by risk levels is based on previous research: Fonseca-Pedrero (42) proposed a typology composed of six levels of progressive latent risk, while other authors have proposed a division into three groups (43), with the aim of comparatively analyzing the effect of trauma and parental socialization styles in relation to the severity of psychotic symptoms. The proportion of participants exceeding the ADHD symptom cut-off was higher in the three at-risk groups than in the control group. Similarly, a closer look at the overall scores on the ADHD Rating Scale questionnaire (inattentive, hyperactive–impulsive, and combined symptom presentations) as we move from the controls to low-, intermediate-, and at-risk individuals, revealed a gradually increasing score for ADHD symptoms. This is consistent with previous studies, which established that 20% of the population with psychosis (15) or ARMS (21) present ADHD throughout their lifetime, as well as other work that found that childhood ADHD is associated with a higher risk of psychotic experiences in adolescence (44).

The developmental stage of the participants should be considered when interpreting these findings. Psychotic-like experiences and attenuated positive symptoms are relatively common and often transient during adolescence, reducing their clinical specificity compared with adult populations (45). Consistent with this, transition rates are generally lower in younger samples, and the predictive value of psychosis-risk indicators may depend more strongly on contextual factors such as risk enrichment (46). These considerations further highlight the importance of incorporating multi-informant assessments, including caregiver reports, when evaluating psychosis risk in adolescents. Although psychosis-risk experiences identified in community-based adolescents may differ from those observed in help-seeking populations (20), our findings indicate that they are associated with greater psychopathology, supporting the clinical relevance of early identification beyond specialized settings.

We did not find any previous studies comparing ADHD subtypes among ARMS subjects. In our study, 68.4% (*n* = 13) of adolescents with an ARMS exceeded the cut-off point for the combined ADHD symptom presentation, compared to 15.8% with the inattentive symptom subtype. This contrasts with the findings published for psychotic disorders in the adult population, which establish more homogeneous percentages between subtypes: 15% (inattentive subtype), 15% (combined subtype), and 10% (hyperactive–impulsive subtype) (15). In other words, the hyperactivity–impulsivity component is much greater in individuals with an ARMS and incipient psychosis than in those with established psychosis. In this sense, our study showed that the presence of hyperactivity–impulsivity but not inattention, specifically among females, can predict belonging to the ARMS group. This relationship between hyperactivity-impulsivity and ARMS is robust because it is significant in both regression and Structural Equation Modeling (SEM), explains a significant proportion of the variance, and the effect size is moderate.

There are many theories that try to explain the association between ADHD and psychosis. First, a possible polygenic contribution has been suggested, as well as the presence of common perinatal complications, which could link ADHD in childhood with the development of schizophrenia in adulthood (12, 14). Secondly, there is a dopaminergic dysregulation in the mesolimbic and mesocortical regions in both pathologies (47). Other theories suggest that treatment for ADHD based on amphetamine derivatives may act as a trigger for positive psychotic symptoms (48). However, only 2.9% of participants in our sample were receiving this treatment, most of whom belonged to the control group; only four individuals in the risk groups were taking stimulant medication, making it unlikely that medication exposure influenced the present findings. Third, several studies show that early adversities such as childhood trauma cause cognitive biases and dopaminergic dysregulation, contributing to the development of both ADHD and psychotic symptoms (24, 49). Finally, cognitive problems such as difficulties with attention and executive functions are frequently observed in both ADHD and psychotic disorders, which can lead to diagnostic confusion (50). That is, early cognitive symptoms typical of psychosis can be misinterpreted as ADHD (50). This confusion is especially common during the clinically at-risk mental state (ARMS), in which symptoms are not yet clear or specific, and before the person develops a full-blown psychotic disorder, when the symptoms may resemble those of ADHD (51).

Although ADHD and ARMS share both internalizing (anxiety, depression, and suicidal ideation) and externalizing symptoms (impulsivity and conduct problems) (52), previous literature has mainly emphasized the role of internalizing symptoms in ARMS populations. In our sample, however, externalizing symptoms also showed a relevant association with ARMS risk. A plausible explanation is that these patients with elevated ADHD symptoms may present difficulties in frustration tolerance, delay of gratification, and emotional regulation, which can trigger disruptive behaviors under stress (24). Additionally, this vulnerability can cause deficits in executive functions, with a consequent deterioration of judgment and critical thinking, making it difficult to evaluate the veracity of some apparently irrational ideas or perceptions and facilitating the emergence and consolidation of anomalous perceptual experiences (53). These interpretations should be considered cautiously and as a hypothesis integrating ADHD and ARMS literature, as studies examining the combined role of internalizing and externalizing symptoms in this context remain scarce.

A key finding of this study is the inverse sex pattern observed in ARMS risk, whereby externalizing symptoms (hyperactivity–impulsivity) predicted psychosis risk in women, whereas in men it was primarily associated with internalizing variables such as depression and suicidal ideation. This finding suggests a sex-specific expression of the association between ADHD symptoms and ARMS, diverging from the typical internalizing profile described in females. Previous literature, largely based on general population simples, indicates that women with ADHD are more likely to present emotional impairment and internalizing symptoms, including anxiety, depression, and suicidal ideation (2, 54), while men more often exhibit externalizing behaviors and motor hyperactivity (21, 26). A possible explanation for our divergent findings is that, in a general population sample, the coexistence of ADHD and psychotic prodromes may alter the typical sex-related symptom pattern, making the usually subtler and under-recognized hyperactivity–impulsivity in women (e.g., cognitive or verbal impulsivity) (55) more clinically evident and associated with ARMS, thereby reflecting a specific vulnerability profile. Nevertheless, these associations did not remain significant in SEM analyses. Overall, our findings support a direct, but not moderating, effect of sex on ARMS and suggest that sex differences shape both ADHD symptoms presentation and its link to emerging psychopathology, highlighting the need for sex-sensitive screening and early intervention approaches that account for differential internalizing and externalizing profiles.

This study has several limitations. Most measures relied on self-reports, which may have led to under- or overestimation, although parent-reported sociodemographic data and CAARMS interviews were used for high-risk participants to reduce bias. Importantly, ADHD symptoms were assessed using the ADHD-RS-IV without clinical diagnostic confirmation. Nonetheless, several studies have demonstrated the reliability of self-reports in adolescent populations (40). The cross-sectional design of the study also prevents any causal relationships or a temporal order between the factors analyzed (ADHD symptoms, ARMS, and sex) from being established and does not allow analysis of whether hyperactivity-impulsivity in women is a precursor to ARMS or an early symptom of it. Furthermore, the community-based, non-help-seeking nature of the sample may imply a lower transition risk than in clinical ARMS populations, which should be considered when interpreting the findings. In addition, the relatively high proportion of invited students who did not participate may have affected the representativeness of the final sample. Although the reasons for non-participation were not systematically assessed, it is possible that willingness to participate was influenced by individual psychopathology, family perceptions of mental health, or other sociodemographic factors, potentially resulting in an under- or over-representation of adolescents with certain clinical or psychosocial characteristics.

Another limitation was the differences in sample sizes between the groups, since, as expected, the higher the risk of psychosis, the lower its prevalence in the general population. To reduce this bias, the Bonferroni correction was applied. It is also worth noting that, although a goodness-of-fit index of less than 0.08 is considered acceptable (56), other authors are more demanding in this regard (57). The exclusion of adolescents with cognitive difficulties or poor proficiency in Spanish—factors potentially linked to at-risk mental states—also represents a methodological limitation. However, only three students were excluded for this reason. Finally, the exclusion of seven potential ARMS cases due to the refusal of their families to complete the CAARMS interview was also a limitation.

## Conclusions

This study provides empirical evidence of an association between ADHD symptoms and at-risk mental states (ARMS) in a non-clinical adolescent population. Combined presentation ADHD symptoms were associated with an increased risk of attenuated psychotic symptoms, supporting its potential role as an early marker of psychotic vulnerability. In addition, ADHD symptom severity increased in parallel with higher ARMS risk levels, suggesting a dimensional relationship between both constructs.

Sex differences emerged as a central finding. The association between ADHD symptoms and ARMS was primarily observed in females and was driven by hyperactivity–impulsivity. In females, hyperactivity–impulsivity and emotional problems predicted ARMS, whereas in males depression and suicidal ideation were the main predictors. Overall, these findings highlight the importance of gender-sensitive early detection strategies and support the need for longitudinal studies to clarify the mechanisms underlying the ADHD–ARMS relationship.

## Data Availability

The raw data supporting the conclusions of this article will be made available by the authors, without undue reservation.
