# Supplementary material for: Clinical overlap between at-risk mental states and attention-deficit/hyperactivity symptoms in community adolescents: investigating the influence of sex
Source: Front Psychiatry. 2026 Sep 23;17:1872849. doi: 10.3389/fpsyt.2026.1872849 (PMC13643517; doi:10.3389/fpsyt.2026.1872849)
Supplement: Supplementary file 1 [file Table1.docx]

**SUPPLEMENTARY MATERIAL**

**Table A.** ADHD Questionnaire Items: ADHD Rating Scale IV

| ADHD RATING SCALE (ITEM) |  | OVERALL SAMPLE  %  (*n*) | Controls  %  (*n*) | Low-risk  %  (*n*) | Intermediate-risk  %  (*n*) | High-risk (ARMS)  %  (*n*) | ${\boldsymbol{\chi}^{\boldsymbol{2}}}$  *p*-value | Effect size (ES)  Corrected typified residuals (CTR) |
| --- | --- | --- | --- | --- | --- | --- | --- | --- |
| 1. When doing my work I fail to pay attention to details or I make mistakes because I am not careful. | Never | 26%  (179) | **33.3%**  **(137)** | **16.2%**  **(38)** | 13%  (3) | **5.3%**  **(1)** | **74.199**  **< 0.001***** | **0.189**  **5.3/−4.2/−2.1**  **−5.1/3.2/4.2**  **−3.3/3.1** |
|  | Sometimes | 55%  (379) | 55.6%  (229) | 57%  (134) | 30.4%  (7) | 47.4%  (9) |  |  |
|  | Frequently | 13.8%  (95) | **8.3%**  **(34)** | **19.6%**  **(46)** | **43.5%**  **(10)** | 26.3%  (5) |  |  |
|  | Very frequently | 5.2%  (36) | **2.9%**  **(12)** | 7.2%  (17) | 13%  (3) | **21.1%**  **(4)** |  |  |
| 2. I wiggle my hands or feet or constantly move in my seat. | Never | 18.4%  (127) | **24.5%**  **(101)** | **9.4%**  **(22)** | 8.7%  (2) | 10.5%  (2) | **59.822**  **< 0.001***** | **0.170**  **5/−4.4**  **3.3/−2.4**  **-**  **−6.2/5/2.2** |
|  | Sometimes | 28.3%  (195) | **33%**  **(136)** | **22.6%**  **(53)** | 13%  (3) | 15.8%  (3) |  |  |
|  | Frequently | 26%  (179) | 23.8%  (98) | 28.9%  (68) | 30.4%  (7) | 31.6%  (6) |  |  |
|  | Very frequently | 27.3%  (188) | **18.7%**  **(77)** | **39.1%**  **(92)** | **47.8%**  **(11)** | 42.1%  (8) |  |  |
| 3. I have difficulty sustaining my attention on tasks or fun activities. | Never | 41.4%  (285) | **53.4%**  **(220)** | **26.4%**  **(62)** | **8.7%**  **(2)** | **5.3%**  **(1)** | **117.136**  **< 0.001***** | **0.238**  **7.8/−5.7/−3.2/−3.2**  **-**  **−6.0/3.6/4.1/3.1**  **−5.2/3.3/4.1** |
|  | Sometimes | 36.9%  (254) | 35.4%  (146) | 40.9%  (96) | 30.4%  (7) | 26.3%  (5) |  |  |
|  | Frequently | 16.4%  (113) | **9.5%**  **(39)** | **23.4%**  **(55)** | **47.8%**  **(11)** | **42.1%**  **(8)** |  |  |
|  | Very frequently | 5.4%  (37) | **1.7%**  **(7)** | **9.4%**  **(22)** | 13%  (3) | **26.3%**  **(5)** |  |  |
| 4. I get up from my seat in situations where I am expected to remain seated. | Never | 68.8%  (474) | **75.7%**  **(312)** | **59.6%**  **(140)** | **47.8%**  **(11)** | 57.9%  (11) | **39.812**  **< 0.001***** | **0.139**  **4.8/−3.8/−2.2**  **-**  **−3.0/2.4**  **−4.3/3.0/2.2** |
|  | Sometimes | 21.6%  (149) | 19.2%  (79) | 25.1%  (59) | 30.4%  (7) | 21.1%  (4) |  |  |
|  | Frequently | 7%  (48) | **4.6%**  **(19)** | **10.2%**  **(24)** | 13%  (3) | 10.5%  (2) |  |  |
|  | Very frequently | (2.6%)  (18) | **0.5%**  **(2)** | **5.1%**  **(12)** | 8.7%  (2) | **10.5%**  **(2)** |  |  |
| 5. I don’t pay attention when spoken to directly. | Never | 61%  (420) | **71.1%**  **(293)** | **48.9%**  **(115)** | **30.4%**  **(7)** | **26.3%**  **(5)** | **71.762**  **< 0.001***** | **0.186**  **6.7/−4.7/−3.1/−3.1**  **−4.7/4.1**  **−4/2.5/3.3**  **2.5/3.0** |
|  | Sometimes | 28.9%  205 | **23.1%**  **(95)** | **39.6%**  **(93)** | 34.8%  (8) | 47.4%  (9) |  |  |
|  | Frequently | 5.8%  (40) | **2.9%**  **(12)** | **8.9%**  **(21)** | **21.7%**  **(5)** | 10.5%  (2) |  |  |
|  | Very frequently | 3.5%  (24) | 2.9%  (12) | (2.6%)  (6) | **13%**  **(3)** | **15.8%**  **(3)** |  |  |
| 6. I feel restless. | Never | 40.8%  (281) | **52.9%**  **(218)** | **25.5%**  **(60)** | **8.7%**  **(2)** | **5.3%**  **(1)** | **102.385**  **< 0.001***** | **0.223**  **7.9/−5.9/−3.2/−3.2**  **−2.2/2.4**  **−3.8/2.5/2.9**  **−5.4/3.1/2.4/4.6** |
|  | Sometimes | 36.9%  (254) | **33.5%**  **(138)** | **43%**  **(101)** | 47.8%  (11) | 21.1%  (4) |  |  |
|  | Frequently | 14.1%  (97) | **10%**  **(41)** | **18.7%**  **(44)** | 21.7%  (5) | **36.8%**  **(7)** |  |  |
|  | Very frequently | 8.3%  (57) | **3.6%**  **(15)** | **12.8%**  **(30)** | **21.7%**  **(5)** | **36.8%**  **(7)** |  |  |
| 7. I do not follow instructions from start to finish and I do not complete the assigned work. | Never | 62.6%  (431) | **71.6%**  **(295)** | **51.9%**  **(122)** | **30.4%**  **(7)** | **36.8%**  **(7)** | **61.093**  **< 0.001***** | **0.172**  **6.0/−4.2/−3.2/2.3**  **−3.5/2.8**  **−3.6/2.5/2.6**  **−2.6/3.6/2.5** |
|  | Sometimes | 27.7%  (191) | **22.8%**  **(94)** | **34.5%**  **(81)** | 34.8%  (8) | 42.1%  (8) |  |  |
|  | Frequently | 7.5%  (52) | **4.6%**  **(19)** | **11.1%**  **(26)** | **21.7%**  **(5)** | 10.5%  (2) |  |  |
|  | Very frequently | 2.2%  (15) | **1%**  **(4)** | (2.6%)  (6) | **13%**  **(3)** | **10.5%**  **(2)** |  |  |
| 8. I have difficulty carrying out activities in my free time in a calm manner or doing fun things quietly. | Never | 54.7%  (377) | **65.3%**  **(269)** | **41.3%**  **(97)** | **34.8%**  **(8)** | **15.8%**  **(3)** | **74.942**  **< 0.001***** | **0.190**  **6.8/−5.1/−2.0/−3.5**  **−3.2/3.2**  **−4.5/3.3/2.6**  **−2.8/3.0/3.5** |
|  | Sometimes | 31.3%  (216) | **26.7%**  **(110)** | **39.1%**  **(92)** | 30.4%  (7) | 36.8%  (7) |  |  |
|  | Frequently | 9.4%  (65) | **5.3%**  **(22)** | **14.5%**  **(34)** | 17.4%  (4) | **26.3%**  **(5)** |  |  |
|  | Very frequently | 4.5%  (31) | **2.7%**  **(11)** | **5.1%**  **(12)** | 17.4%  (4) | **21.1%**  **(4)** |  |  |
| 9. I have difficulty organising tasks and activities. | Never | 39.6%  (273) | **48.1%**  **(198)** | **28.5%**  **(67)** | 21.7%  (5) | **15.8%**  **(3)** | **90.82**  **< 0.001***** | **0.210**  **5.5/−4.3/−2.2**  **-**  **−5.0/4.5/2.6**  **−5.3/2.5/4.2/3.9** |
|  | Sometimes | 38.9%  (268) | 40%  (165) | 38.3%  (90) | 39.1%  (9) | 21.1%  (4) |  |  |
|  | Frequently | 15.7%  (108) | **10%**  **(41)** | **24.3%**  **(57)** | 13%  (3) | **36.8%**  **(7)** |  |  |
|  | Very frequently | 5.8%  (40) | **1.9%**  **(8)** | **8.9%**  **(21)** | **26.1%**  **(6)** | **26.3%**  **(5)** |  |  |
| 10. I feel like I have to ‘continuously move’ or ‘like I’m being driven by a motor’. | Never | 56.2%  (387) | **69.4%**  **(286)** | **39.1%**  **(92)** | **21.7%**  **(5)** | **21.1%**  **(4)** | **92.546**  **< 0.001***** | **0.212**  **8.5/−6.5/−3.4/−3.1**  **−3.1/2.3/2.2**  **−5.0/4.3**  **−4.7/3.1/3.1** |
|  | Sometimes | 24.1%  (166) | **19.9%**  **(82)** | **29.4%**  **(69)** | **43.5%**  **(10)** | 26.3%  (5) |  |  |
|  | Frequently | 12.2%  (84) | **7%**  **(29)** | **19.6%**  **(46)** | 17.4%  (4) | 26.3%  (5) |  |  |
|  | Very frequently | 7.5%  (52) | **3.6%**  **(15)** | **11.9%**  **(28)** | 17.4%  (4) | **26.3%**  **(5)** |  |  |
| 11. I dislike, avoid, or am reluctant to undertake work that requires sustained mental effort. | Never | 57%  (393) | **69.4%**  **(286)** | **40.4%**  **(95)** | 39.1%  (9) | **15.8%**  **(3)** | **115.215**  **< 0.001***** | **0.236**  **8.0/−6.3/−3.7**  **−3.0/3.1/2.1**  **−6.5/5.0/2.8**  **−3.8/5.0** |
|  | Sometimes | 30.3%  (209) | **26%**  **(107)** | **37.9%**  **(89)** | 13%  (3) | **52.6%**  **(10)** |  |  |
|  | Frequently | 9.3%  (64) | **3.4%**  **(14)** | **17%**  **(40)** | **26.1%**  **(6)** | 21.1%  (4) |  |  |
|  | Very frequently | 3.3%  (23) | **1.2%**  **(5)** | 4.7%  (11) | **21.7%**  **(5)** | 10.5%  (2) |  |  |
| 12. I talk excessively. | Never | 28%  (193) | **34.2%**  **(141)** | **19.1%**  **(45)** | 26.1%  (6) | **5.3%**  **(1)** | **45.186**  **< 0.001***** | **0.148**  **4.4/−3.7/−2.2**  **−2.8/2.9**  **−4.3/2.9/3.4** |
|  | Sometimes | 37.6%  (259) | 39.6%  (163) | 34.9%  (82) | 30.4%  (7) | 36.8%  (7) |  |  |
|  | Frequently | 19.4%  (134) | **16%**  **(66)** | **25.5%**  **(60)** | 21.7%  (5) | 15.8%  (3) |  |  |
|  | Very frequently | 14.9%  (103) | **10.2%**  **(42)** | **20.4%**  **(48)** | 21.7%  (5) | **42.1%**  **(8)** |  |  |
| 13. I lose things that are not necessary to carry out tasks or activities. | Never | 48.3%  (333) | **61.2%**  **(252)** | **31.5%**  **(74)** | **26.1%**  **(6)** | **5.3%**  **(1)** | **103.932**  **< 0.001***** | **0.224**  **8.2/−6.4/−2.2/−3.8**  **−3.4/3.2/2.0**  **−4.2/3.2/2.4**  **−5.3/3.0/3.7/3.2** |
|  | Sometimes | 35.7%  (246) | **30.6%**  **(126)** | **43.8%**  **(103)** | 26.1%  (6) | **57.9%**  **(11)** |  |  |
|  | Frequently | 10.9%  (75) | **6.8%**  **(28)** | **16.2%**  **(38)** | **26.1%**  **(6)** | 15.8%  (3) |  |  |
|  | Very frequently | 5.1%  (35) | **1.5%**  **(6)** | **8.5%**  **(20)** | **21.7%**  **(5)** | **21.1%**  **(4)** |  |  |
| 14. I answer abruptly before others finish asking me the question. | Never | 55.3%  (381) | **65.8%**  **(271)** | **40.9%**  **(96)** | **34.8%**  **(8)** | **31.6%**  **(6)** | **130.82**  **< 0.001***** | **0.252**  **6.7/−5.5/−2.0/−2.1**  **2.3/7.1**  **−4.4/5.7/4.9** |
|  | Sometimes | 31.3%  (216) | 29.9%  (123) | 34.9%  (82) | 17.4%  (4) | 36.8%  (7) |  |  |
|  | Frequently | 10.6%  (73) | **3.9%**  **(16)** | **20.9%**  **(49)** | 26.1%  (6) | 10.5%  (2) |  |  |
|  | Very frequently | 2.8%  (19) | **0.5%**  **(2)** | 3.4%  (8) | **21.7%**  **(5)** | **21.1%**  **(4)** |  |  |
| 15. I get distracted easily. | Never | 22.1%  (152) | **28.6%**  **(118)** | **13.2%**  **(31)** | **4.3%**  **(1)** | 10.5%  (2) | **114.943**  **< 0.001***** | **0.236**  **5.1/−4/−2.1**  **4.1/−2.7/−2.6**  **−9.2/7.0/2.0/5.0** |
|  | Sometimes | 38.9%  (268) | **45.1%**  **(186)** | **31.9%**  **(75)** | 21.7%  (5) | **10.5%**  **(2)** |  |  |
|  | Frequently | 20%  (138) | 18.4%  (76) | 21.3%  (50) | 39.1%  (9) | 15.8%  (3) |  |  |
|  | Very frequently | 19%  (131) | **7.8%**  **(32)** | **33.6%**  **(79)** | **34.8%**  **(8)** | **63.2%**  **(12)** |  |  |
| 16. I have difficulty waiting my turn. | Never | 48.2%  (332) | **57%**  **(235)** | **37.4%**  **(88)** | **21.7%**  **(5)** | **21.1%**  **(4)** | **82.497**  **< 0.001***** | **0.200**  **7.7/−4.1/−2.6/−2.4**  **−2.9/2.6/2.6**  **−5.3/2.5/2.9/5.3** |
|  | Sometimes | 32.5%  (224) | 30.8%  (127) | 35.3%  (83) | 26.1%  (6) | 42.1%  (8) |  |  |
|  | Frequently | 12.5%  (86) | **9.5%**  **(39)** | **17%**  **(40)** | **30.4%**  **(7)** | 0%  0 |  |  |
|  | Very frequently | 6.8%  (47) | **2.7%**  **(11)** | **10.2%**  **(24)** | **21.7%**  **(5)** | **36.8%**  **(7)** |  |  |
| 17. I am forgetful in daily activities. | Never | 42.5%  (293) | **51.7%**  **(213)** | **31.9%**  **(75)** | **13%**  **(3)** | **10.5%**  **(2)** | **61.799**  **< 0.001***** | **0.173**  **5.9/−4.1/−2.9/−2.9**  **−3.2/2.6**  **−4.9/3.6/2.6** |
|  | Sometimes | 34.3%  (236) | 32.8%  (135) | 36.2%  (85) | 30.4%  (7) | 47.4%  (9) |  |  |
|  | Frequently | 15.5%  (107) | **11.9%**  **(49)** | 19.1%  (45) | **34.8%**  **(8)** | 26.3%  (5) |  |  |
|  | Very frequently | 7.7%  (53) | **3.6%**  **(15)** | **12.8%**  **(30)** | **21.7%**  **(5)** | 15.8%  (3) |  |  |
| 18. I interrupt others or am intrusive. | Never | 63.6%  (438) | **75.2%**  **(310)** | **48.1%**  **(113)** | **34.8%**  **(8)** | **36.8%**  **(7)** | **74.014**  **< 0.001***** | **0.189**  **7.8/−6.1/−2.9/−2.5**  **−4.6/3.5**  **−4.9/4.3**  **−3.2/2.1/2.2** |
|  | Sometimes | 27.9%  (192) | **21.4%**  **(88)** | **36.2%**  **(85)** | 43.5%  (10) | 47.4%  (9) |  |  |
|  | Frequently | 6.4%  (44) | **2.7%**  **(11)** | **11.9%**  **(28)** | 13%  (3) | 10.5%  (2) |  |  |
|  | Very frequently | 2.2%  (15) | **0.7%**  **(3)** | **(3.8%)**  **(9)** | **8.7%**  **(2)** | 5.3%  (1) |  |  |

*Note* : Significant differences are highlighted in bold. **p* < 0.05; ***p* < 0.01; ****p* < 0.001.
